# Young Age at Diagnosis of Type 1 Diabetes Is Associated with the Development of Celiac Disease—Associated Antibodies in Children Living in Newfoundland and Labrador, Canada

**DOI:** 10.3390/children2040403

**Published:** 2015-10-14

**Authors:** Harpreet Pall, Leigh A. Newhook, Hillary Aaron, Joseph Curtis, Ed Randell

**Affiliations:** 1 Department of Pediatrics, Drexel University College of Medicine and St. Christopher’s Hospital for Children, Philadelphia, PA 19134, USA; E-Mail: hillary.aaron@drexelmed.edu (H.A.); 2 Janeway Pediatric Research Unit, Faculty of Medicine, Memorial University of Newfoundland, St. John’s, NL A1B 3V6, Canada; E-Mails: lnewhook@mun.ca (L.A.N.); anthonyjoe.curtis@gmail.com (J.C.); 3 Department of Laboratory Medicine, Memorial University of Newfoundland, St. John’s, NL A1B 3V6, Canada; E-Mail: ed.randell@easternhealth.ca (E.R.)

**Keywords:** Celiac disease, pediatrics, type 1 diabetes

## Abstract

Objectives: The objectives of this study were to establish the prevalence of positive antibodies to endomysium (EMA) and tissue transglutaminase (tTG) in children with type 1 diabetes living in Newfoundland and Labrador (NL), and to examine clinical features associated with positive antibodies. Methods: Patients were recruited from the pediatric diabetes clinic. One hundred sixty-seven children with type 1 diabetes from the 280 children followed at the clinic were prospectively screened for celiac disease using EMA and tTG. The variables of Irish descent, age at onset of diabetes, duration of diabetes, sex, family history of celiac disease, hemoglobin A1C (A1C), ferritin, gastrointestinal symptoms, and body mass index were compiled for all patients. The group of patients with positive antibodies to EMA and/or tTG was compared to the group with negative antibodies. Results: The prevalence of patients with positive antibodies to EMA and/or tTG was 16.8% (*n* = 28). One patient had also been previously diagnosed with symptomatic celiac disease. The two statistically significant variables with positive antibodies were an earlier age at onset of diabetes (Mann-Whitney U two-tailed test: mean difference 3.2 years, 95% CI 1.7–4.8 years, *p* < 0.0001) and longer duration of diabetes (Mann-Whitney U two-tailed test: mean difference 2.9 years, 95% CI 1.3–4.4 years, *p* < 0.0001). Irish descent was associated with positive antibodies but did not reach statistical significance. On logistic regression analysis performed with these three variables together, only age at onset of diabetes remained significant. Conclusions: There is a high prevalence of celiac disease-associated antibodies in children living in NL with type 1 diabetes. Unlike other clinical features, an earlier age at onset of diabetes was predictive for positive antibodies. As the majority of children with positive antibodies did not have signs or symptoms of celiac disease, routine screening for celiac disease in type 1 diabetes is recommended.

## 1. Introduction

Celiac disease (CD) is an immune-mediated disease caused by sensitivity to gluten in genetically susceptible individuals. It is both common and underdiagnosed. Signs and symptoms are multiple, diverse, and non-specific. They range from classic chronic diarrhea and failure to thrive to simple iron deficiency anemia. Most patients with CD have very few typical symptoms of malabsorption. It is hypothesized that these asymptomatic patients are also at risk of developing other autoimmune diseases and malignancies if the diagnosis is delayed and a gluten-free diet not initiated [1].

The co-existence of type 1 diabetes with CD was first suspected in 1954 and diagnosed by small intestinal biopsy in 1969. It has now been widely accepted for decades that the prevalence of CD is increased in patients with type 1 diabetes. According to studies in the recent literature, the prevalence of CD in patients with type 1 diabetes ranges from 4.4%–12.4% compared to a 1% prevalence in the general population [1,2,3,4]. A recent publication also found the prevalence of CD to be 16% in first-degree relatives of patients with type 1 diabetes [5]. It is hypothesized that this increased association is due to a common genetic basis. In both diseases, genetic susceptibility is associated with the Human Leukocyte Antigen (HLA) DQ2-DQ8 genotype, as well as non-HLA genes [1,6]. Many diabetes clinics now routinely screen for CD in their patients, though consensus-based guidelines offer disparate recommendations in terms of screening: The American Diabetes Association and the North American Society for Pediatric Gastroenterology, Hepatology and Nutrition recommend screening all children with a diagnosis of type 1 diabetes for CD regardless of symptoms, while the Canadian Diabetes Association and an National Institutes of Health (NIH) sponsored consensus document recommend screening only symptomatic children with type 1 diabetes for CD [7,8,9,10]. The recently published guidelines from the International Society of Pediatric and Adolescent Diabetes (ISPAD) recommend that screening for CD should be performed at the time of diabetes diagnosis, and every 1–2 years thereafter. More frequent assessment is indicated if the clinical situation suggests the possibility of CD or the child has a first-degree relative with CD [11].

The endomysial (EMA) and transglutaminase (tTG) immunoglobulin (Ig)A antibody tests are routinely used to screen for CD. Numerous studies have evaluated the accuracy of these tests in both the general population and in groups at risk. The sensitivity of the EMA in children ranges from 0.88–1.00 and specificity ranges from 0.91–1.00 [7,12]. The sensitivity of the tTG in children ranges from 0.92–1.00, and the specificity from 0.91–1.00 [7,12]. The tTG test, done by enzyme immunoassay technique, has the advantage of having less inter-observer variability compared with the EMA test, done by immunofluorescence assay using a microscope.

Newfoundland and Labrador (NL) is a unique province in Canada with an island portion (Newfoundland) and a mainland portion (Labrador), and due to relative isolation and a founder population some genetic disorders are more prevalent. The Avalon Peninsula in Newfoundland is well defined geographically and contains 46% of the childhood population of Newfoundland. A recent study determined the incidence of type 1 diabetes in the 0–14 years age group in the Avalon Peninsula to be one of the highest in the world (49.9 per 100, 000 per year, with a linear increase over the study period of 1.03 per 100, 000 per year) [13]. Suspected reasons for such a high incidence of type 1 diabetes in NL include shared genetic background of its inhabitants, as 96% of residents have ancestors from Ireland or southwest England. In addition there are environmental factors such as northern latitude and vitamin D deficiency, low breastfeeding rates, and high rates of caesarean section [13], which could contribute to the high incidence. The prevalence of CD in NL has not yet been studied. It has been shown that Ireland has the highest prevalence of CD in the world and as mentioned previously, Irish descendants comprise a significant proportion of the founder population in NL [14].

The Janeway Child Health Care Centre (JCHCC) is the only tertiary care children’s hospital servicing the province of NL. All children with type 1 diabetes who live on the Avalon Peninsula are referred to one of the pediatric diabetologistsat the JCHCC and are followed by the Janeway Pediatric Diabetes Team. Based on our population demographics, we postulated a high prevalence of CD-associated EMA and tTG antibodies in children followed for type 1 diabetes at the JCHCC.

The specific aims were to: (1) determine the cross-sectional prevalence of CD-associated EMA and tTG antibodies in a cohort of children with type 1 diabetes; and (2) determine whether hemoglobin A1C (A1C), serum ferritin, age at onset of diabetes, duration of diabetes, gastrointestinal symptoms, body mass index, sex, Irish descent, and family history of CD are useful in predicting presence of CD-associated EMA and tTG antibodies in children with type 1 diabetes.

## 2. Materials and Methods

This study examined cross-sectionally positive antibodies to EMA and tTG in a cohort of children with type 1 diabetes and no history of CD. Ethics approval was obtained from the Human Investigations Committee of Memorial University. There were approximately 280 children (0–18 years) who were followed at the Diabetes Clinic at the JCHCC during the time frame of this study. One patient, not of Irish descent, had been previously diagnosed with symptomatic biopsy proven CD prior to this study. A total of 175 patients were recruited to this study from the Diabetes Clinic between November 2000 and October 2001.

Inclusion criteria for this study included: (1) type 1 diabetes; (2) Age 0–18 years; (3) Diagnosis of type 1 diabetes based on the Canadian Diabetes Association Clinical Practice Guidelines [8]. The exclusion criteria were parental refusal or refusal from child of legal consenting age, and other forms of diabetes (*i.e.*, Type 2 diabetes, Cystic-fibrosis related diabetes, *etc.*). If the child fitted the study criteria, our diabetes research nurse approached the family and asked if they wanted to participate. Information concerning the study and CD with its implications was then given, and written informed consent obtained.

Height and weight were routinely measured at each appointment in the Diabetes Clinic on a stadiometer. Body mass index (BMI) was calculated for each patient. Information concerning the date the child was diagnosed with diabetes was obtained from the Diabetes Database. Parents were asked about a family history of CD and Irish descent. A detailed pedigree for each family was also collected. The parents were also asked whether the child was having any symptoms suggestive of CD (diarrhea, flatulence, abdominal distension, abdominal pain, vomiting).

All children entered into the study were screened for CD using both IgA based EMA and tTG antibodies along with their routine biochemistry (A1C). The last four A1C values (when available) were averaged. A1C was measured on an A1C 2.2 Glycohemoglobin analyzer (Tosoh, King of Prussia, PA, USA); Serum ferritin was measured on the Access Immunoassay System (Beckman-Coulter, Brea, CA. USA); IgA was measured an Array Immunochemistry system (Beckman-Coulter). Presence of IgA EMA was determined by a commercially available kit based on indirect immunofluorescence microscopy using fixed cryostat sections of monkey esophagus (Scimedx, Densville, NJ, USA.) as an antigen substrate. Patient sera were initially diluted 1:20 and subsequently investigated at dilutions of 1:40, 1:80, 1:160, 1:320, and 1:640. The antibody titer was reported as the highest serum dilution yielding fluorescence. Performance characteristics of this kit were previously investigated by others [15]. Positivity for EMA antibody was defined at a cut-off titer of 1:20 or greater. The tTG assays were performed at the Barbara Davis Centre for Childhood Diabetes, University of Colorado Health Sciences Centre (the results are given based on absorbance with an index 0.05 defined as positive). Age specific reference ranges were used to identify patients with low levels of IgA. Patients with low IgA levels were screened for CD using IgG anti-reticulin antibodies by immunofluorescence assay (Scimedx).

The group with positive antibodies to EMA and/or tTG was compared to the group with negative antibodies. Univariate analysis was performed between the positive and negative groups on all variables. Mann-Whitney U two-tailed test was used for continuous independent variables, and chi-square test was used for dichotomous independent variables. Those variables statistically significant were then entered into a logistic regression model. The statistical software package SPSS was used for all statistical analysis.

If the child was EMA and/or tTG antibody positive, the family was given information on CD and referred to a pediatric gastroenterologist for possible small bowel biopsy to make a confirmatory diagnosis of CD.

## 3. Results

A total of 175 patients were recruited from the JCHCC. Results on CD-associated antibodies were available on 167 patients. All patients included in analysis had a history of type 1 diabetes. Mean age was 12.4 years with a range of 2.6 to 18.9 years. Gender was evenly split with 49.7% of patients being male (Table 1).

**Table 1:** Descriptive statistics of subjects with type 1 diabetes and their CD-associated antibodies.

Participants (*n* = 167, Plus 1 Patient with Previously Diagnosed CD)	Characteristic
Mean age years (range)	12.4 (2.6–18.9)
Male gender	83 (49.7%)
	**Antibody status (%)**
Positive EMA-IgA only	2 (1.2)
Positive tTG-IgA only	15 (9.0)
Positive EMA-IgA and tTG-IgA	11 (6.6)
Positive EMA-IgA and/or tTG-IgA	28 (16.8)
Both antibodies negative	139 (83.2)

(CD = celiac disease; EMA = antibody to endomysium, tTG = antibody to tissue transglutaminase; IgA = immunoglobulin A).

The prevalence of patients with positive antibodies to EMA and/or tTG was 16.8% (*n* = 28) (Table 1). In addition to these 28 patients, 1 patient had been previously diagnosed with symptomatic CD. Thirteen patients had high titers of tTG defined as >0.575 (median value for positive results). Eight patients had high titers of EMA defined as titer >1:160. All patients with low IgA levels (*n* = 6) were negative for anti-reticulin IgG antibodies. When comparing patients with positive antibodies to those with negative, ferritin, gender, A1C, Irish descent, family history of celiac disease, gastrointestinal symptoms, and BMI were not associated with positive antibodies (Table 2). The two statistically significant variables with positive antibodies were an earlier age at onset of diabetes (Mann-Whitney U two-tailed test: mean difference 3.2 years, 95% CI 1.7–4.8 years, *p* < 0.0001) and longer duration of diabetes (Mann-Whitney U two-tailed test: mean difference 2.9 years, 95% CI 1.3–4.4 years, *p* < 0.0001). Irish descent was associated with positive antibodies but did not reach statistical significance. On logistic regression analysis performed with these three variables together, only age at onset of diabetes remained significant.

**Table 2:** Comparison of subjects with positive EMA-IgA and/or tTG-IgA *versus* those negative for these antibodies.

	EMA-IgA and tTG-IgA Negative	EMA-IgA and/or tTG-IgA Positive	*p* Value
Age at onset of diabetes in years (SD)	8.2 (3.8)	5.0 (3.8)	*p* < 0.0001
Duration of diabetes in years (SD)	4.2 (3.6)	7.1 (4.3)	*p* < 0.0001
Irish descent	55.4%	71.4%	
Family history of celiac disease	2.9%	3.6%	
Male gender	51.8%	39.3%	
Positive gastrointestinal symptoms	25.2%	17.9%	
BMI (SD)	21.3 (4.8)	20.6 (5.1)	
A1C % (SD)	8.7 (1.4)	9.2 (1.0)	
Low ferritin	12.9 %	21.4%	

(BMI (body mass index) = weight (kg)/[height (m)]^2^; A1C = hemoglobin A1C; SD = standard deviation).

## 4. Discussion

These results suggest one of the highest prevalence of CD-associated antibodies in children with type 1 diabetes [1,2,3,4]. This is noteworthy since complications of untreated CD include malabsorption, short stature, failure to thrive, iron deficiency anemia, osteopenia/osteoporosis, delayed puberty, and even malignancy.

The high incidence of CD-associated antibodies in children with type 1 diabetes in this population may also be due in part to the strong inherent autoimmune predisposition with specific HLA alleles associated with both diseases. The incidence of type 1 diabetes in NL is one of the highest in the world [13]. HLA DQ2-DQ8 heterozygotes show a high risk for type 1 diabetes development, and are also strongly associated with CD. The high risk genotype HLA DQ2-DQ8 is also associated with young age at onset of diabetes [16,17]. Data about HLA genotypes was not available, which is a weakness of this study. Similarities have been shown between the mechanisms of recognition of gluten by HLA molecules in CD and of pancreatic autoantigens in type 1 diabetes [6]. Up to one third of those with type 1 diabetes with HLA DQ2 have positive tTG, compared with less than 2% of those without HLA DQ2 or DQ8 [18]. Genome-wide association studies have also revealed numerous non-HLA CD-associated loci, and sixty-four percent of these loci are shared with at least one other autoimmune disease, further emphasizing a common genetic predisposition [6].

Our results also revealed that earlier age at onset of diabetes was predictive for positive CD antibodies. These findings are consistent with those of other studies in that patients with both type 1 diabetes and CD are characterized by a younger onset of type 1 diabetes [3,19]. This is in agreement with the suggestion that there are common genetic and environmental factors that contribute to the etiology and pathogenesis of these diseases in younger children. There was no other characteristic that was significantly able to predict the presence of CD-associated EMA and tTG antibodies in these children.

A drawback to this study is the absence of confirmatory biopsies in these patients. The antibody tests are screening tests, and not a basis on which to make a definitive diagnosis of CD. The criteria for a diagnosis of CD must include histology with the characteristic features. The positive predictive value (PPV) of serologic screening for biopsy evidence of disease in children considered genetically at risk for CD (type 1 diabetes, first-degree relative, at-risk HLA type on newborn screening) has been quoted at 0.73–0.80. This is lower than the PPV reported for patients who are identified on the basis of symptoms, which approaches 1.00. Thus, the prevalence of biopsy-proven CD in children living in NL with type 1 diabetes is likely lower [20]. The prevalence of CD in the general NL population is not known, and this is also a limitation.

Another limitation of this study is that the data was collected over a decade ago. At the time of data collection, in the early 2000s, the association between type 1 diabetes and CD was just coming to light. Patients with positive antibodies were referred to pediatric gastroenterology, however data from that follow-up is unavailable. Currently, it is widely accepted that there is a genetic association between the two diseases, and most children with a diagnosis of type 1 diabetes are routinely screened for CD. Nonetheless, our results reveal one of the highest prevalence of CD-associated antibodies in children with type 1 diabetes [1,2,3,4].

The practice of screening for CD at JCHCC has been based on the current Canadian Diabetes Association guidelines [8]. CD screening has been performed when there are recurrent gastrointestinal symptoms, poor linear growth, poor weight gain, fatigue, anemia, unexplained frequent hypoglycemia or poor metabolic control. All patients with positive antibodies have been referred to pediatric gastroenterology for confirmatory endoscopy with biopsy. However, the main purpose of screening asymptomatic children is to prevent long-term complications of undiagnosed CD. Our data supports screening all children with type 1 diabetes.

## 5. Conclusions

The results presented in this manuscript strongly support the current ISPAD recommendations (11). In our population, earlier age at onset of diabetes was predictive for positive CD antibodies. CD associated antibodies should be screened at the time of diagnosis of type 1 diabetes, and at 1–2 year intervals thereafter, or even more frequently if there is a positive family history of CD.

## References

[1] Camarca M.E., Mozzillo E., Nugnes R., Zito E., Falco M., Fattorusso V., Mobilia S., Buono P., Valerio G., Trocone R. (2012). Celiac disease in type 1 diabetes mellitus. Riv. Ital. Pediatr..

[2] Tsouka A., Mahmud F.H., Marcon M.A. (2015). Celiac Disease Associated with Type 1 Diabetes and Celiac Disease Alone: Are These Patients Different?. J. Pediatr. Gastroenterol. Nutr..

[3] Cerutti F., Bruno G., Chiarelli F., Lorini R., Meschi F., Sacchetti C. (2004). Younger age at onset and sex predict celiac disease in children and adolescents with type 1 diabetes. Diabetes Care.

[4] Hansson T., Dahlbom I., Tuvemo T., Frisk G. (2015). Silent coeliac disease is over-represented in children with type 1 diabetes and their siblings. Acta Paediatr..

[5] Bakker S.F., Tushuizen M.E., Stokvis-Brantsma W.H., Aanstoot H.J., Winterdijk P., van Setten P.A., von Blomberg B.M., Mulder C.J., Simsek S. (2013). Frequent delay of coeliac disease diagnosis in symptomatic patients with type 1 diabetes mellitus: clinical and genetic characteristics. Eur. J. Intern. Med..

[6] Trocone R., Discepolo V. (2014). Celiac Disease and Autoimmunity. J. Pediatr. Gastroenterol. Nutr..

[7] Hill I.D., Dirks M.H., Liptak G.S., Colletti R.B., Fasano A., Guandalini S., Hoffenberg E.J., Horvath K., Murray J.A., Seidman E.G. (2005). Guidelines for the Diagnosis and Treatment of Celiac Disease in Children: Recommendations of the North American Society for Pediatric Gastroenterology, Hepatology and Nutrition. J. Pediatr. Gastroenterol. Nutr..

[8] Canadian Diabetes Association Clinical Practice Guidelines Expert Committee (2013). Canadian diabetes association 2013 clinical practice guidelines for the prevention and management of diabetes in Canada. Can. J. Diabetes.

[9] American Diabetes Association (2009). Standards of Medical Care in Diabetes—2009. Diabetes Care.

[10] (2004). NIH Consensus Development Conference on Celiac Disease. NIH Consens. State Sci. Statements.

[11] Kordonouri O., Klingensmith G., Knip M., Holl R.W., Aanstoot H.J., Menon P.S., Craig M.E. (2014). International Society for Pediatric and Adolescent Diabetes. ISPAD Clinical Practice Consensus Guidelines 2014. Other complications and diabetes-associated conditions in children and adolescents. Pediatr. Diabetes.

[12] Sud S., Marcon M., Assor E., Palmert M.R., Daneman D., Mahmud F.H. (2010). Celiac Disease and Pediatric Type 1 Diabetes: Diagnostic and Treatment Dilemmas. Int. J. Pediatr. Endocrinol..

[13] Newhook L.A., Penney A., Fiander J., Dowden J. (2012). Recent incidence of type 1 diabetes mellitus in children 0–14 years in Newfoundland and Labrador, Canada climbs to over 45/100,000: A retrospective time trend study. BMC Res. Notes.

[14] West J., Fleming K.M., Tata L.J., Card T.R., Crooks C.J. (2014). Incidence and Prevalence of Celiac Disease and Dermatitis Herpetiformis in the UK over Two Decades: Population-Based Study. Am. J. Gastroenterol..

[15] Hansson T., Dahlbom I., Rogberg S., Dannaeus A., Hopfl P., Gut H., Kraaz W., Klareskog L. (2002). Recombinant human tissue transglutaminase for diagnosis and follow-up of childhood coeliac disease. Pediatr. Res..

[16] Karjalainen J., Salmela P., Ilonen J., Surcel H.M., Knip M. (1989). A comparison of childhood and adult type I diabetes mellitus. N. Engl. J. Med..

[17] Caillat-Zucman S., Garchon H.J., Timsit J., Assan R., Boitard C., Djilali-Saiah I., Bougnères P., Bach J.F. (1992). Age-dependent HLA genetic heterogeneity of type 1 insulin-dependent diabetes mellitus. J. Clin. Investig..

[18] Bao F., Yu L., Babu S., Wang T., Hoffenberg E.J., Rewers M., Eisenbarth G.S. (1999). One third of HLA DQ2 homozygous patients with type 1 diabetes express celiac disease-associated transglutaminase antibodies. J. Autoimmun..

[19] Kaspers S., Kordonouri O., Schober E., Grabert M., Hauffa B.P., Holl R.W. (2004). Anthropometry, Metabolic Control and Thyroid Autoimmunity in Type 1 Diabetes with Celiac Disease: A Multicenter Survey. J. Pediatr..

[20] Hoffenberg E.J., Bao F., Eisenbarth G.S., Uhlhorn C., Haas J.E., Sokol R.J., Rewers M. (2000). Transglutaminase antibodies in children with a genetic risk for celiac disease. J. Pediatr..

